# The Effect of Extrinsic Factors on the Mechanical Behavior and Structure of Elastic Dental Ligatures and Chains

**DOI:** 10.3390/polym14010038

**Published:** 2021-12-23

**Authors:** Kata Csekő, Péter Maróti, Zsuzsanna Helyes, Roland Told, Fanni Riegler, József Szalma, Zsuzsanna Gurdán

**Affiliations:** 1 Department of Pharmacology and Pharmacotherapy, Medical School & Szentágothai Research Centre, University of Pécs, Ifjusag Str. 20, H-7624 Pecs, Hungary; csekoe.kata@gmail.com (K.C.); zsuzsanna.helyes@aok.pte.hu (Z.H.); 2 3D Printing and Visualization Centre, University of Pécs, Boszorkany Str. 2, H-7624 Pecs, Hungary; told.roland@pte.hu; 3 Medical Simulation Education Centre, Medical School, University of Pécs, Szigeti Str. 12, H-7624 Pecs, Hungary; 4 Department of Oral and Maxillofacial Surgery, University of Pécs, 5 Dischka Gy Str., H-7621 Pecs, Hungary; riegler.fanni@pte.hu (F.R.); szalma.jozsef@pte.hu (J.S.); 5 Department of Paediatric and Adolescent Dentistry, Medical School, University of Pécs, 5 Dischka Gy Str., H-7621 Pecs, Hungary

**Keywords:** elastomers, orthodontics, extrinsic factors, dental ligatures, dental chains, force degradation, force decay, mechanical testing, scanning electron microscopy

## Abstract

Force provided by elastomers used in orthodontics can be affected by several factors present in the oral cavity. The aim of our study was to investigate the role of mouthwashes, toothbrushing, and smoking in the force decay of such elastomers. Tensile strength, changes in the force continuously exerted, and force decay of elastic chains (Ortho Organizer and Masel Short Power Chain) and elastic ligatures (Dentaurum and Masel) by two separate manufacturers were measured. Measurements were initially made on untreated elastics, followed by exposure to different environmental factors including cigarette smoke, toothbrushing (mechanical plaque control), and two different mouthwashes (chemical plaque control). Changes on the surface of the elastics were studied with scanning electron microscopy (SEM). Untreated Masel elastic ligature showed lower tensile strength than Dentaurum elastic ligature (2339 cN vs. 3660 cN), while significantly higher tensile strength was measured for Ortho Organizer elastic chains than Masel chains (2639 cN vs. 1324 cN). The decrease in the elastic force of Masel ligature was greater in response to all external factors compared to Dentaurum. Although brushing with toothpaste and toothbrush impacted the force of both Masel and Ortho organizer ligatures negatively, force degradation was more apparent in the case of the Ortho organizer. Surface changes were more visible when applying Curasept mouthrinse, however force decay was higher in the Corsodyl group. Mechanical and chemical plaque control can influence the tensile strength and force decay of orthodontic elastomers, which should be considered by selecting the elastomers or determining their changing interval for the practice.

## 1. Introduction

Elastic ligatures and chains are used in different clinical indications during orthodontic treatment. Such indications can be correction of midline deviations, treatment of malocclusions, closure of diastema or extra spaces due to tooth extraction, space closure, canine distalization, correction of tooth rotation, and the attachment of the archwire to the brackets [1,2]. Their high flexibility, resistance to external forces, low price, as well as their easy handling by the patients make them essential appliances in orthodontic practice. However, despite their ability to endure relatively high forces, studies show that force degradation decreases their efficacy over time, which can be considered their major limitation [3]. Therefore, characterizing the force decay of elastomers, especially due to external factors attributed to chemical and mechanical plaque control, is of utmost importance for optimizing therapeutic guidelines.

Force degradation can be influenced by several factors, although the composition and diameter of the elastomer undoubtedly determines its endurance the most. Often the only available data on the chemical composition of elastomers is their latex content. Studies however show slightly controversial force decay results when comparing latex and non-latex elastomers [4,5,6]. Other important factors that can influence force decay include saliva, pH, temperature changes, enzymatic and microbial events, the duration of the extension, as well as chemical plaque control [7,8,9]. Mechanical degradation is deemed to be the main reason behind force degradation in clinical practice. Variety in the resistance of seemingly same materials can be due to morphologic and dimensional variances, differences in manufacturing technology, or even additives added to the rubber base. The wet environment, enzymes found in the oral cavity, temperature fluctuation, and the traction itself also greatly affect their mechanical properties [10].

A frequently used material of elastomers in orthodontics is polyurethane (PU), a petroleum-derived polymer. The enzymatic degradation of PU, similar to other petro-polymers, occurs in two stages. The first stage is adsorption of enzymes on the elastomer surface then hydro-peroxidation/hydrolysis of the bonds [11]. These degrading enzymes can be found in the digestive tract of some invertebrates and in some microorganisms as well, such as *P. aeruginosa*, *Corynebacterium* sp., *Comamonas acidovorans*, *Pseudomonas fluorescens*, *Acinetobacter calcoaceticus*, and *Bacillus subtilis* [11].

In human, degrading enzymes can be originating from the salivary glands, inflammatory responses, microorganisms, and mononuclear phagocytic cells [12]. Oral cavity enzymes are classified as carbohydrates, esterases, catalases and oxidases, proteolytic enzymes such as proteinase, and others such as carbonic anhydrase [13]. In addition, monocyte-derived macrophages may also secrete hydrolytic enzymes [14]. Two PUase enzymes have been isolated and characterized so far. One is a cell associated, membrane bound PU-esterase, while the other is a soluble, extracellular PU-esterase [15]. Cholesterol esterase and carboxyl esterase are very effective in PU degradation, while some serine proteases had smaller degradative capacity [14]. The above-mentioned biodegradation can be measured by substrate weight loss, changes in the mechanical properties and/or the chemical structure [11].

The resistance to tension of elastic modules is of crucial importance for the clinician since it influences the length of the treatment [16]. In some studies, force degradation was greater in wet than in dry environments, so artificial saliva provided the medium during force degradation studies [17,18]. Force degradation studies evaluating the effect of pH suggest that to polyurethane chain elasticity inversely correlates to the pH of the mouth, basic pH levels being the most hostile [19]. Previous studies have shown that latex-free elastomeric ligatures induced less cell lysis than latex-containing ones. The cytotoxicity of orthodontic elastomeric ligatures is not a negligible consideration as they are in very close contact with the gums and oral mucosa [20]. According to a recent study, non-latex elastics deform to a greater extent during use after 24 h of treatment time [21]. Due to the increasing hypersensitivity reactions to latex-containing materials, the use of non-latex, polyurethane materials are necessary and widespread. Synthetic materials can replace latex products if they are replaced with sufficient frequency [22].

Given that optimal exertion is an essential part of successful orthodontic treatment, it is necessary to understand force reduction and the factors that affect it. This knowledge will help clinicians plan appropriate intervals between orders to activate or replace the transmission unit (elastomer chains/ligatures) and modify the influencing factors to maintain optimal orthodontic force during treatment. This comparative study also helps clinicians understand the accuracy and clinical reliability of evidence from in vitro studies on the subject.

There is no consensus on either the replacement frequency of elastics to achieve the most effective treatment, or which criteria should the dentist rely on when choosing between products of different manufacturers for different treatment options. It is not known which external factors influence the effectivity of elastic chains and how long they can truly exert enough force to facilitate tooth movement, so the clinician can achieve maximal effect with minimal chair time [23,24,25,26].

In the present study we aimed to investigate the maximal load capacity of two elastic ligatures and chains of different manufacturers, as well as to compare their continuous force exertion upon continuous stretching. Furthermore, our scope was to evaluate the effects of chemical and mechanical plaque control of the most common oral care products, such as two different mouthwashes and toothpaste, as well as smoking on the force decay and surface morphology on these dental elastomers.

## 2. Materials and Methods

Two types of elastic ligatures and chains were used during the study: (1) the Dentaurum 6.4 mm (ref. no. 77231600; 1.4 inch) ligature (Dentaurum GmbH & Co. KG, Ispringen, Germany) which is transparent and latex-free; (2) the heavy Masel 7.95 mm (ref. no. 5011404) 5/16 inch) ligature made of natural latex (Masel Enterprises, Bristol, UK); (3) the Ortho organizer short power elastic and transparent chain (ref. no. 400317, Ortho Organizers, Aston, CA, USA); as well as (4) the Masel short power latex free chain (ref. no. 4108538, Masel Enterprises, Bristol, UK). In case of the chains, five chain links have been used to the tests. The original length of the intact Ortho and Masel chains were 14.0 mm and 14.2 mm, respectively.

### 2.1. Tensile Tests

Tensile testing of both types of elastic ligatures and chains was performed by a Zwick/Roell Z5.0 biaxial universal testing machine (ZwickRoell, Ulm, Germany) on room temperature (23 °C ± 1 °C), using 3-3 pieces of each. The tests were done according to the ISO 37:2011 guideline. Ligatures were tested as a ring-shape, and chains were approximated as a dumbbell shape because the cross-section of the chain can be considered constant across its full length due to its design. Elastic chains and ligatures were fixed to the grips with a steel ring with a thickness of 1.2 mm. Throughout the measurements the preload was 20 cN, and crosshead speed was set to 500 mm/min in the case of the ligatures and 200 mm/min at chains.

### 2.2. Force Degradation Measurement

**Figure 1:**
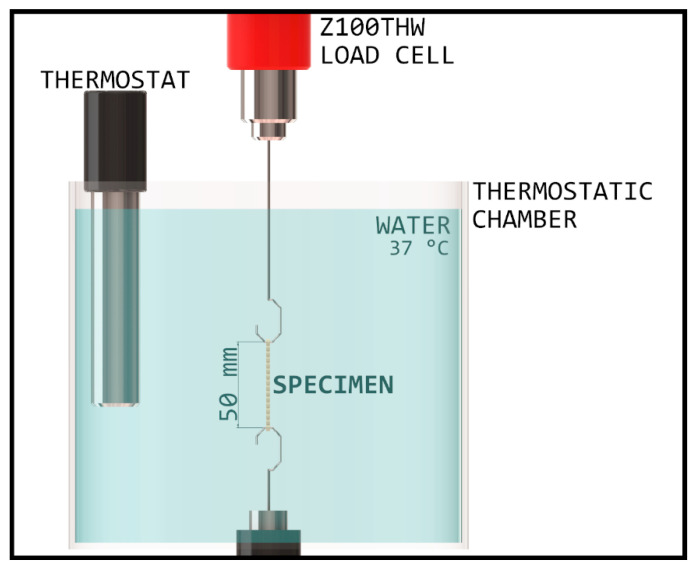
Schematic diagram of the thermostatic chamber, with an established environment corresponding to the oral cavity.

After tensile strength testing, force degradation was measured. The oral environment was simulated with a thermostatic chamber and waterbath, wherein the temperature was set to 37 °C ± 2 °C. The chamber was built inside the Zwick/Roell Z100THW Universal material tester with 1 kN load cell (Figure 1).

The specimens were attached to two hooks with 1 mm cross section diameter followed by 5 min of acclimation time, so the whole specimen would be heated to the set temperature before extension. Next, they were stretched to 50 mm and kept at this length for 24 h, during which tensile force was recorded and displayed on a diagram by the testXpert III program of testing machine. In favor of temperature stability, the uppermost part of the elastic was submerged into water to at least 50 mm. The measurements were repeated on three different test specimens with every material, respectively.

### 2.3. Experimental Protocol for Measuring Force Degradation Due to Extrinsic Factors

**Figure 2:**
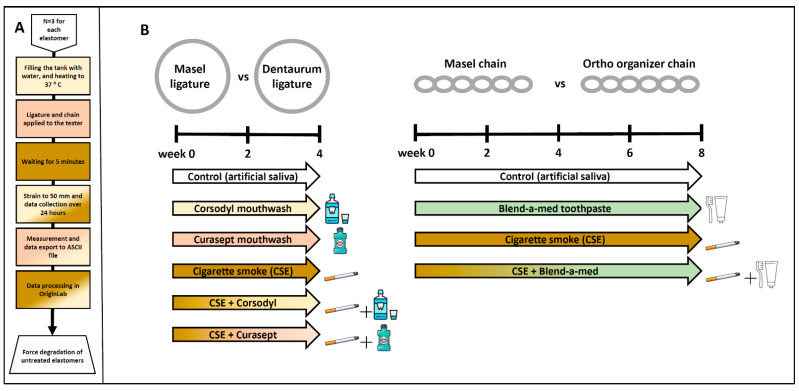
(**A**) Schematic diagram of force degradation measurement protocol, in case of untreated elastomers. (**B**) Experimental protocol for measuring force degradation of Masel and Dentaurum ligatures, as well as Masel and Ortho organizer chains due to chemical and mechanical plaque control.

We investigated the effects of various plaque control treatments on force decay of elastic ligatures and chains of different manufacturers (Figure 2). The effect chemical plaque control with Corsodyl (in the study marked as Cors; Corsodyl, GlaxoSmithKline, Brentford, UK), and Curasept (in the study, marked as Cura; Curasept ADS 205, Curaden Healthcare S.p.A., Saronno, Italy) mouthwashes combined with or without cigarette smoke exposure (in the study, marked as CSE) was studied on elastic ligatures (Masel and Dentaurum) for 4 weeks, while the effect of mechanical plaque control with Blend-a-med toothpaste (in the study, marked as Blend; Blend-a-med Pro-Expert All-in-One, Procter & Gamble, Gross-Gerau, Germany) and toothbrush (Curaprox CS 1560; Curaprox, Kriens, Switzerland)) combined with or without cigarette smoke exposure was studied on elastic chains (Masel and Ortho Organizer) for 8 weeks. At the start of the experiment elastomers were placed in 6-well plastic plates (*n* = 6/treatment group/well). 3 mL of artificial saliva was pipetted into each well and changed daily. The artificial saliva was prepared with 1 L of distilled water, 0.4 g of sodium chloride, 0.4 g of potassium chloride, 0.906 g calcium chloride dihydrate, 0.69 g sodium dihydrogene phosphate dehydrate, 0.005 g sodium sulfide nanohydrate, and 1 g urea.

Elastic ligatures (*n* = 6/manufacturer/treatment group) were rinsed with either (1) Corsodyl, an alcohol-free menthol flavored mouthwash with 0.2% chlorhexidine gluconate as an active ingredient or (2) Curasept (Curasept ADS 205, Curaden Healthcare S.p.A., Saronno, Italy) mouthwash containing 0.05% chlorhexidine digluconate and 0.05% sodium fluoride. The elastic ligatures from the corresponding groups were placed in 1 mL mouthwash pipetted freshly into Eppendorf tubes and shaken for 1 min for 5 days/week, mimicking mouth rinsing. Next, the elastomers were placed onto a filter paper, the mouthwash was blotted, and they were placed back into the 6-well plate with artificial saliva pipetted freshly onto them. The groups were subdivided into cigarette smoke-exposed (Cors + CSE or Cura + CSE) or non-exposed (Cors or Cura) groups (for CSE description see below). Ligatures placed in artificial saliva were used as control specimens.

The elastic chains were cleansed with Blend-a-med toothpaste containing fluoride and abrasives. Curaprox CS 1560 soft strength manual toothbrush was used to mimic mechanical plaque control for 3 min, once daily. Imitating cleaning of the upper denture, the toothbrush was held at a 45° angle and applied with circular motions without pressing. Next, the toothpaste was rinsed off with water, surplus liquid was blotted from the elastomers, and they were placed back into their respective wells on the 6-well plate and had fresh artificial saliva pipetted onto them. The groups were subdivided into cigarette smoke-exposed (Blend + CSE) or non-exposed (Blend) groups (for CSE description see below). Chains placed in artificial saliva were used as control specimens.

Both types of elastic ligatures and chains were exposed to chronic cigarette smoke (CSE group) (3R4F research cigarette, Kentucky Tobacco Research and Development Center, University of Kentucky, Lexington, KY, USA) for 30 min, twice a day, 5 days a week for the duration of 4 or 8 weeks in the case of ligatures and chains, respectively, with the help of a TE-2 two port cigarette smoke exposure chamber (Teague Enterprise, Woodland, CA, USA). Following cigarette smoke exposure (CSE), the elastomers were placed in fresh artificial saliva each day. Subgroups of all chemical and mechanical plaque control groups were also exposed to CSE (see above).

### 2.4. Force Degradation Measurement Due to Extrinsic Factors

**Figure 3:**
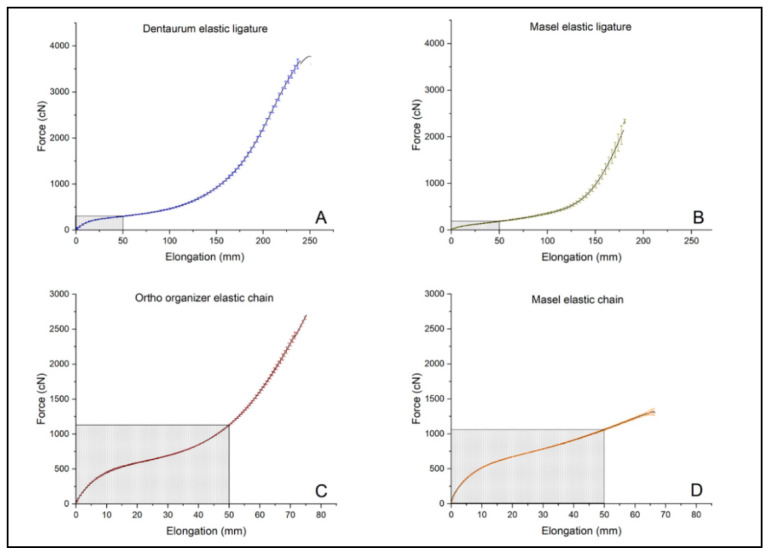
Tensile strength of untreated, intact (**A**) Dentaurum elastic ligature, (**B**) Masel elastic ligature, (**C**) Ortho organizer elastic chain, (**D**) Masel elastic chain. Shaded rectangles indicate the range of their general usage based on clinical experience (*n* = 3/elastomer).

The strength of the elastic chains was measured in cN at the beginning, and every two weeks after treatment by a manual force gauge (Dentaurum GmbH, Ispringen, Deutschland) with the help of a pin fixed to a ruler. The measuring range of the gauge was 98–980 cN (Figure 3). In order to have better comparison on the force decay between the elastomers of different manufacturers upon chronic exposure to certain extrinsic factors we calculated force degradation in % as well, by taking the initially measured strength values (in cN) as 100% and calculating the % values of respective elastomers at later time points.

### 2.5. Scanning Electron Microscopy Imaging

At the end of the experimental protocol, scanning electron microscopy was performed to examine surface changes with a JSM-6300 JEOL Scanning Microscope (SEMTechSolutions, North Billerica, MA, USA) equipped with a digital camera and an INCAx-sight (Oxford Instruments, Abingdon, UK) element analyzer with 20,000 V acceleration voltage. Gold sheeting has been applied to the test samples.

### 2.6. Statistics

Force degradation curve fitting and offset calculations were done with the OriginPro 2018 program (OriginLab Corporation, Northampton, MA, USA). Upon fitting the curve to the series of data points, two exponential decay functions were observed. However, in order to make the evaluation easier by excluding the data from the first 4 h (which were determined by logarithmizing of the measured curves and plotting the start of the second straight line), where one of the exponentials was dominant, only one exponential decay curve was fitted to the data points from every measurement using the following equation:

$$y\left(t\right)=y_{0}+Ae^{\frac{-t}{\tau }},$$

where *y*_0_—offset of the function, *A*—constant amplitude of the function, *τ*—time constant of the exponential function, *t*—value of x axis, which indicates time in this case. The 24 h and 21 days results at the force degradation measures were compared by two-sample *t*-test. 21 day results were extrapolated from the measured data. The level of significance was set as *p* ≤ 0.05. The normality of data was tested using the Kolmogorov–Smirnov test. Comparison of groups exposed to different extrinsic factors was done with two-way ANOVA, Sidak, and Tukey post hoc tests using GraphPad Prism v8 software (GraphPad Software, San Diego, CA, USA). The level of significance was set at *p* ≤ 0.05.

## 3. Results

### 3.1. Tensile Test

As the primary objective of the test was not only to characterize the material, but to determine its suitability for use in dentistry, the maximum force (cN) and elongation (mm) were measured according to the dental methodology, rather than tensile strength (MPa) and deformation (%). Results of tensile test in case of intact, untreated ligatures, and chains: ${\bar{F}}_{max}$—mean of the maximum force (cN), σ_F_—standard deviation of ${\bar{F}}_{max}$ (cN), ${\bar{E}}_{M}$—elongation at ${\bar{F}}_{max}$ (mm) and σ_EM_—standard deviation of ${\bar{E}}_{M}\;$(mm) shown in Table 1. The highest values have been measured in case of the Dentarum ligature elastic (${\bar{F}}_{max}$ = 3660.4 cN ± 167.3 cN), while the highest elongation at maximum force was measured with the Ortho organizer chain (${\bar{E}}_{M}\;$= 449.6 mm ± 14.4 mm) The force-elongation diagrams can be seen in Figure 3.

### 3.2. 24 h Interval Force Degradation Measurement of Intact, Untreated Elastomers in a Thermostatic Chamber

The elastomers were placed in a thermostatic chamber set to 37 °C. The force degradation was continuously measured for 24 h. The Dentaurum elastic ligatures showed uniform force levels, the values calculated for the 21st day did not change significantly, but its variance increased significantly. Difference of the forces of the Masel elastic ligatures was already significant at 24 h, and since the R2 (COD; coefficient of determination) values of the fitted exponential curve were between 0.68–0.9, further force values (21st day) could not be calculated. In the other cases, the values of R2 were higher than 0.98. Masel and Ortho elastic chains showed consistent outcomes during force degradation measurements in the thermostatic chamber. The results of the measurements are indicated in Table 2.

### 3.3. Force Degradation of Dentaurum and Masel Elastic Ligatures Exposed to Extrinsic Factors

**Figure 4:**
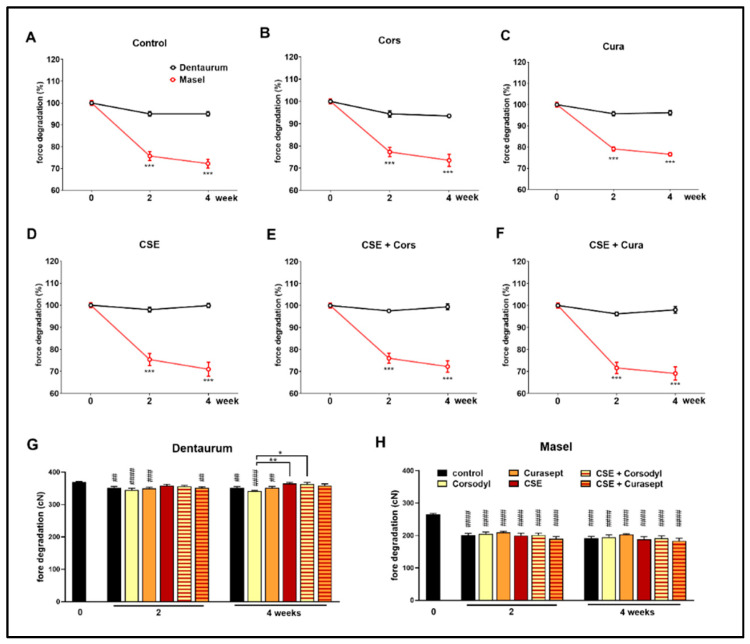
Comparison of percentages of force decay of (**A**) untreated control, (**B**) Corsodyl, (**C**) Curasept, (**D**) cigarette smoke (CS), (**E**) CSE + Corsodyl, and (**F**) CSE + Curasept treated Dentaurum (black) and Masel (red) elastic ligatures at 50 mm extension. *** *p* < 0.0001 vs. Dentaurum, (*n* = 6/group; two-way ANOVA and Sidak post hoc tests). Force degradation of (**G**) Dentaurum and (**H**) Masel elastic ligatures to external factors during the 4-week-long protocol. ## *p* < 0.005, ### *p* < 0.0005, #### *p* < 0.0001 vs. intact values; * *p* < 0.05, ** *p* < 0.005 highlightsdifference between two treatments at given time. (*n* = 6/group, two-way ANOVA, and Sidak post hoc tests).

Elastic ligatures from different manufacturers (the Masel ligature containing latex and the latex-free Dentaurum) were rinsed with Curasept and Corsodyl 1 min/day, 5 days/week for 4 weeks to mimic chemical plaque control, as well as exposed to cigarette smoke in combination with or without the mouthwash treatment (Figure 2). The force of the control Dentaurum elastic ligatures immersed in artificial saliva decreased significantly by the end of the 2nd and 4th weeks has been compared to its intact value measured before the experiment (Figure 4G), however, the force degradation was minimal in this case compared to the Masel elastomer (Figure 4A). Similar force decay was observed in both the Cors group (*p* < 0.0001 at both measured times) and the Cura group (2nd week *p* < 0.0005; 4th week *p* < 0.005), as well as compared to the initial value. Meanwhile, force of the Dentaurum elastics exposed to cigarette smoke did not decrease significantly (Figure 4G). No significant differences were found in the statistical results comparing the different treatments up to the 4th week. Elastics degrade more as a result of Corsodyl treatment than due to combined exposure to cigarette smoke (*p* = 0.0008). Congruent with the force degradation measurement extrapolated to day 21, the Masel ligatures immersed in artificial saliva showed a remarkable force decrease by the end of the 2nd and the 4th weeks compared to the initial values (*p* < 0.0001). Similar force degradations were measured in all groups, there was no apparent difference between different treatments (Figure 4H). The absolute force value exerted by the Masel ligature was substantially lower than that of the Dentaurum ligature. Moreover, this lower initial value was followed by a considerably greater force decay already on the 2nd week in every Masel group (Figure 4A–F). These data support the greater resistance of the Dentaurum elastic ligature. Although it shows significant force loss with time in certain groups (control, Cors group, Cura group on weeks 2 and 4), this is negligible compared to the Masel elastic.

### 3.4. Force Degradation of Ortho Organizer and Masel Elastic Chains Exposed to Extrinsic Factors

**Figure 5:**
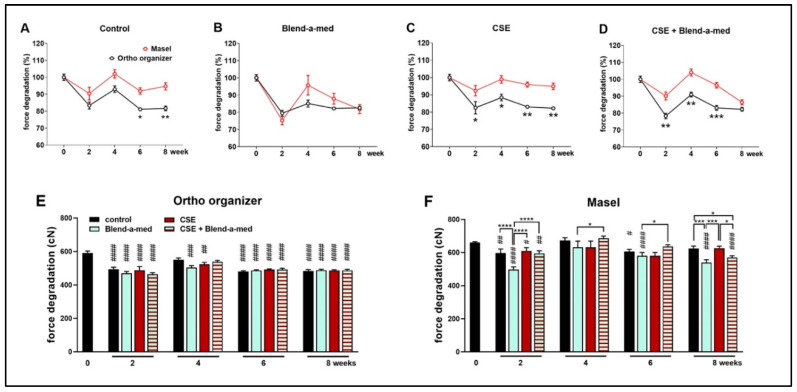
Percentages of force degradation of (**A**) control, untreated; (**B**) Blend-a-med; (**C**) cigarette smoke and (**D**) cigarette smoke + Blend-a-med treated Ortho organizer (black) and Masel (red) elastic chains at 50 mm extension. Force degradation of (**E**) Ortho organizer and (**F**) Masel elastic chains to different external factors in cN *n* = 6/group; two-way ANOVA and Sidak post hoc tests, * *p* < 0.05, ** *p* < 0.005, *** *p* = 0.0005 vs. Masel. Two-way ANOVA and Tukey post hoc tests. # *p* < 0.05, ## *p* < 0.005, ### *p* < 0.0005, #### *p* < 0.0001, vs. intact values; * *p* < 0.05, *** *p* < 0.0005, **** *p* < 0.0001 difference between two treatments at given timepoint.

Ortho organizer and Masel elastic chains were cleaned with Blend-a-med toothpaste and brushed 1 min/day, for 5 days/week for 4 weeks in order to mimic mechanical plaque control, as well as exposed to cigarette smoke in combination with or without cleaning (Figure 2). The control, untreated Ortho organizer chain had similar force as the Masel chain in intact conditions (591.37 cN ± 54.3 cN vs. 660 cN ± 26.4 cN, respectively) (Figure 5E,F). However, the control samples of Masel chains immersed in artificial saliva exhibited greater force consistently throughout the 8-week-long protocol, as opposed to the Ortho organizer, which, after a transient increase at week 4, showed significantly greater force degradation (Figure 5A). Interestingly, there was no significant difference between the 2 types of elastics cleaned with toothpaste (Figure 5B), however, it was apparent that this exogenic factor influenced the Masel elastic more negatively than any other treatments (Figure 5F). Cigarette smoke significantly decreased the force of Ortho organizer (Figure 5E), however, it was not apparent in case of the Masel chain, which was more resistant to CSE (Figure 5F). The force of the Ortho organizer elastic chain was significantly decreased by all treatments, and at all timepoints by approximately 20% compared to the intact value measured at the beginning of the study, with the exception of the control group at week 4 which showed a transient increase. However, there was no significant difference in the force of Ortho organizer chains between the different external factors (Figure 5E). The force of the control Masel elastic chains showed transient alterations, being significantly decreased at weeks 2 and 6, however, the changes were similar to the intact values at week 4 in all groups, and by the end of the protocol in the control and CSE-treated specimens. Interestingly, the elastomers cleaned with toothpaste showed significant force degradation at weeks 2, 6, and 8.

### 3.5. Scanning Electron Microscopy Images

**Figure 6:**
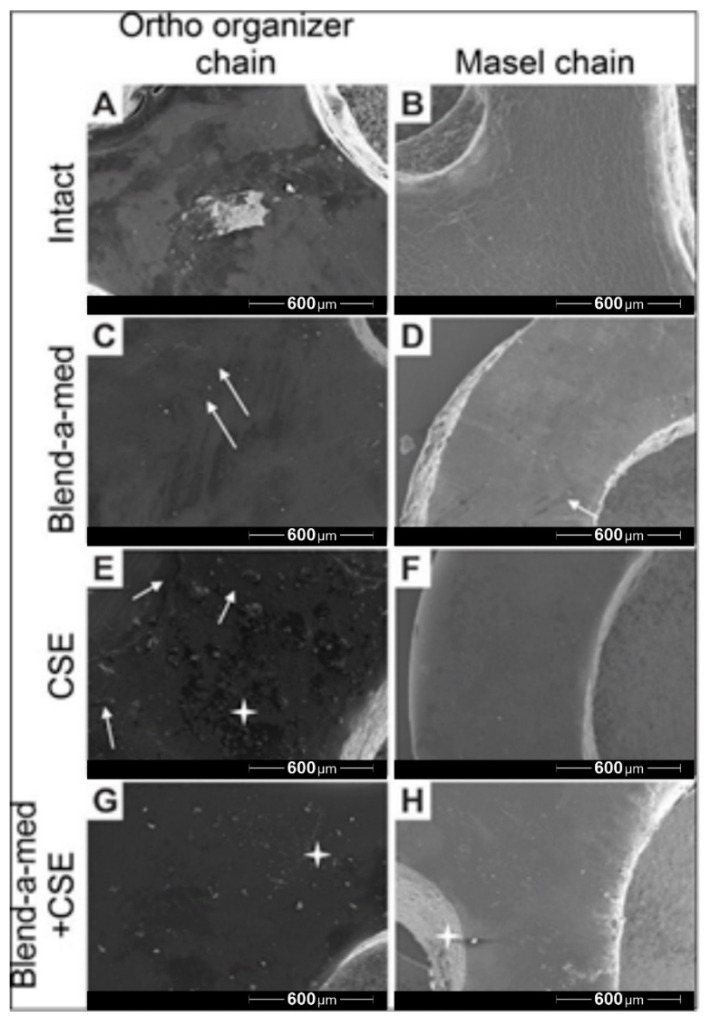
Representative electron microscopy images of (**A**,**C**,**E**,**G**) Ortho Organizer and (**B**,**D**,**F**,**H**) Masel elastic chains after 8 weeks of exposure to (**A**,**B**) control environment (artificial saliva), (**C**,**D**) cleaning with Blend-a-med toothpaste, (**E**,**F**) cigarette smoke as well as (**G**,**H**) cigarette smoke combined with mechanical cleaning with toothpaste. The white arrows indicate the microlaesions, and a white start highlights particles from smoke exposure. 80× magnification.

Scanning electron microscopy (SEM) recordings were captured of the Ortho Organizer (Figure 6 left column) and Masel (Figure 6 right column) chains at the end of the protocol, after 8-week-long exposure to control environment (artificial saliva) (Figure 6A,B), cleaning with Blend-a-med toothpaste (Figure 6C,D), cigarette smoke (Figure 6E,F), as well as cigarette smoke combined with mechanical cleaning with toothpaste (Figure 6G,H). The surface of the Ortho organizer chain was heterogeneous (Figure 6A), with multiple light and darker spots. Presumably, saliva particles adhered to the surface of the elastomers, which was less prominent on the surface of the Masel chain. The surface of the Masel elastomer (Figure 6B) was more homogeneous, however the whole area was seemingly wrinkled. Cleaning with Blend-a-med toothpaste removed surface contaminations, however, toothbrush abrasing the surface created microlesions, through which the toothpaste could penetrate deeper, which was macroscopically observable by the greenish discoloration of the elastomers in the respective groups. Microlesions occurred on the surface of the Masel chain as well (Figure 6D), and at higher magnifications it is visible that the wrinkled surface remains. Smoke particles due to CSE treatment adhered to the surface of the Ortho organizer (Figure 6E), whereas surface contaminations were barely observable on the Masel elastic (Figure 6F) as smoke particles were repelled to a greater extent, the surface was almost perfectly homogeneous. Mechanical plaque control with toothpaste and toothbrush removed most of the smoke particles (Figure 6G,H). Interestingly, more smoke particles stuck onto the Masel elastic chain, despite mechanical cleaning (Figure 6H).

**Figure 7:**
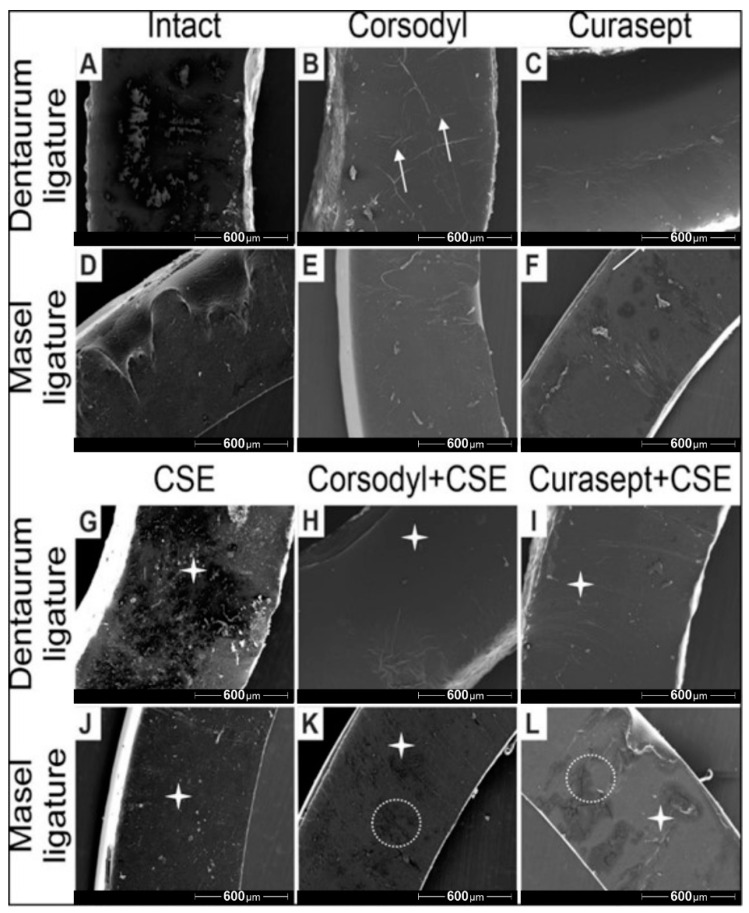
Representative scanning electron microscopy images of Dentarum and Masel ligatures 8 weeks after exposure to (**A**,**D**) artificial saliva (control ligatures), (**B**,**E**) Corsodyl, (**C**,**F**) Curaspet, (**G**,**J**) cigarette smoke, (**H**,**K**) Corsodyl and cigarette smoke, (**I**,**L**) Curasept and cigarette smoke. White arrows represent the microlesions, the white stars mark smoke particles, and the dotted lines indicate the major cracks on the surface of Masel ligatures. 80× magnification.

The SEM imaging also revealed structural changes on the surface of ligatures. Both the Corsodyl and Curasept treatments caused minor cracks and lesions on the surface (Figure 7B,C,E,F), which was more pronounced in case of Corsodyl. Particles from smoke were observable on all ligatures with CSE treatment (Figure 7G,J). The most prevalent degradation and structural damage could be observed on the Masel ligature exposed to Corsodyl and CSE, and to Curasept and CSE (Figure 7K,L).

## 4. Discussion

Space closure is one of the biggest challenges in orthodontic practice and in order to avoid unwanted side effects, one requires a thorough knowledge of biomechanics [27]. Materials suffer from an initial force loss of 40–50% in the first few hours, after which force degradation continues for 2–3 weeks to a lesser extent. These observations are supported by our tension experiments as well. After the decrease in the first 4 h, all of the materials were able to preserve the optimal force (150–200 cN) that is necessary for tooth movement in the long term [28], with the exception of the Masel elastic ligature. Force degradation is caused mainly by stretching itself, the second most important influencing factor is the temperature which cannot be controlled in the oral cavity. Elastics exert lower force on higher temperatures, nevertheless still provide the force guaranteed by the manufacturer [29]. Saliva itself also causes force decay, although to a lesser extent than mechanic tension and it only becomes significant weeks after the start of their application [8]. Means of personal oral hygiene such as mouthwashes, toothpastes, and the scouring effect from toothbrushes also affects the value of force [30]. Due to the ever-increasing latex allergy among patients, use of latex-free elastic chains became widespread during procedures. Synthetic rubbers are plastics that have very similar properties to natural latex. They can be silicones, polyurethanes, or styrene-butadiene rubbers (SBR) [31,32]. Commonly called elastomers, these elastic materials are amorphous polymers containing polyurethane, and are similar to plastic and able to return to their original shape following deformation [10]. On top of avoiding allergic reactions, latex-free elastomers also showed more advantageous mechanical properties [33,34]. Both types of elastic bands tested in our experiment were latex-free, which are commonly used during treatment.

During brushing of the teeth, elastic rubbers used on patients’ fixed braces come in direct contact with abrasive agents and fluoride, which can influence their structure or release of force. Preventing demineralization and decalcification/white spot lesions are important both during and after fixed braces treatment [35,36]. The Blend-A-Med toothpaste which we used in the experiment showed good results regarding fluoride content of the superficial enamel layer [37]. Toothpastes containing 1450 ppm sodium fluoride (NaF) combined with tin (II) chloride (SnCl2) show significantly better results in terms of preventing plaque growth than toothpastes only containing sodium fluoride [38]. In case of the chains, Masel chains were resistant to the effect of toothpaste to a lesser extent, and to other treatments to a greater extent, where their force loss was approximately half compared to that of the Ortho organizer elastic chains, although they teared more easily. Brushing with toothpaste and toothbrush had a negative effect on the force of the chains, regardless they should be cleaned for oral hygiene reasons. For the proper cleaning of a fixed bracket, mouthwash is also necessary on top of a toothbrush, which helps prevent the spread of bacteria [39]. However, most mouthwashes also contain alcohol, dyes, and different aromas besides the beneficial fluoride and chlorhexidine. Due to potential side effects, practitioners recommend the use of alcohol-free mouthwashes, which contain chlorhexidine gluconate, cetylpyridinium chloride, and essential oils as their active components [40,41]. The combination of chlorhexidine and fluoride proved to be the most effective for the prevention of caries associated dental diseases among patients wearing fixed braces. Fewer data are available in the literature about their effect on elastic chains used during fixed bracket treatment. Mouthwashes were proved to be able to change the pH of the oral cavity due to their chemical composition, and thereby influence orthodontic appliances and devices [42]. It was shown that ethanol attaches to polyurethane polymers, therefore a power chain coming into contact with alcohol-containing mouthwash suffers a greater force degradation than those coming into contact with only saliva or alcohol-free mouthwashes [25]. The scanning electron microscopic images supported the findings regarding the force degradation. Smoke exposure clearly affects the structure and integrity of both the ligatures and chains. Combining with mouthwash, the structural damage is even more severe, and they cause surface cracks and lesions. Of course, in case of smoking during orthodontic treatment, its negative impact on health is greater than the negative effect on the chain. The limitation of our study was that we could not combine extrinsic factors and continuous force load during the experiment. Furthermore, we could not standardize the brushing with toothpaste, so that bristles from the toothbrush reach the whole surface of the chain evenly, and this could influence our results as well. Although biodegradation of PU elastomers in the oral cavity is a known process, the artificial saliva used in this study was not able to completely simulate in vivo degradation mechanisms. Later, in order to overcome limitations of the in vitro experiments, it would be worthwhile to evaluate properties of the samples in vivo, so we could investigate and estimate the real performance of the materials in a clinical setting within the oral cavity of the patients.

## 5. Conclusions

Force of elastomers decreases significantly within the first few hours of extension. In our present study we compared the maximum load capacity and strength of two types of ligatures and bands of two different manufacturers by continuous stretching, as well as their force decay upon chemical and mechanical plaque control. Our findings show that Dentaurum non-latex elastic ligature has higher tensile strength and significantly less force decay than the natural latex Masel ligature. Regarding elastic chains, although Ortho organizer proved to have higher tensile strength initially compared to the non-latex Masel chain, our investigation showed better long-term endurance of Masel chains. Interestingly, neither chemical, mechanical plaque control, or smoking influenced the force degradation of the elastomers remarkably.

## Figures and Tables

**Table 1:** The mean values of the maximum force (${\bar{F}}_{max}$), its standard deviation (σ_F_), elongation at ${\bar{F}}_{max}$; $({\bar{E}}_{M}$), and its standard deviation (σ_EM_).

	$\;{\bar{F}}_{max}$ (cN)	*σ*_F_ (cN)	${\bar{E}}_{M}$ (mm)	σ_EM_ (mm)
Dentaurum elastic ligature	3660.4	167.3	241.5	7.8
Masel elastic ligature	2339.1	465.0	182.5	4.9
Ortho organizer elastic chain	2639.6	150.4	74.18	2.38
Masel elastic chain	1324.6	72.9	66.79	3.48

**Table 2:** Force degradation measurements of the samples measured at 24 h timepoint and the calculated values for day 21. The *p* value refers to the difference between the meausrements of the 24th hour, and the extrapolated data on the 21st day.

	Measured Data		Calculated Data		*t*-Test
	${\bar{F}}_{24h}$ (cN)	σ_24h_ (cN)	${\bar{F}}_{21\mathrm{day}}$ (cN)	σ_21day_ (cN)	*p* Value
Dentaurum elastic ligature	171.73	2.5	109.8	36.8	0.10 *
Masel elastic ligature	176.1	17.25	-	-	-
Ortho organiser elastic chain	198.53	3.05	191.8	4.22	0.089 **
Masel elastic chain	197.42	2.28	187.47	1.62	0.0036 **

* Not equal variance. ** Equal variance.

## Data Availability

Data is contained within the article.
